# Revisitando o Uso do CDI na Cardiomiopatia Chagásica: Evidências Atuais e Direções Futuras

**DOI:** 10.36660/abc.20240423

**Published:** 2024-08-07

**Authors:** André Carmo, Antônio Luiz Pinho Ribeiro

**Affiliations:** 1 Universidade Federal de Minas Gerais Faculdade de Medicina Hospital das Clínicas Belo Horizonte MG Brasil Hospital das Clínicas e Faculdade de Medicina da Universidade Federal de Minas Gerais, Belo Horizonte, MG – Brasil

**Keywords:** Doença de Chagas, Cardiomiopatia Chagásica, Insuficiência Cardíaca, Acidente Vascular Cerebral, Cardioversores Desfibriladores Implantáveis/tendências

A cardiomiopatia chagásica (CC) é uma doença cardíaca infecciosa crônica que recentemente foi considerada uma cardiomiopatia arritmogênica infecciosa de acordo com a declaração do HRS sobre o assunto.^1^ Após uma fase aguda, os pacientes tornam-se cronicamente infectados, mostrando uma ampla gama de apresentações relacionadas a doenças cardíacas, incluindo insuficiência cardíaca, acidente vascular cerebral, anomalias de condução, fibrilação atrial e arritmia ventricular.^2^ A morte súbita é uma forma de morte da maioria dos pacientes com CC, e o cardioversor-desfibrilador implantável (CDI) tem sido utilizado como terapia preferencial, pelo menos para prevenção secundária, seguindo recomendações semelhantes às de outras doenças cardiovasculares.^3^

Todas as informações sobre o uso do CDI na CC são obtidas de estudos observacionais e, em relação às taxas de mortalidade, duas metanálises avaliaram esse tema em diferentes populações. A primeira meta-análise, realizada pelo nosso grupo, encontrou taxa de mortalidade anual de 9,7% (IC 95%: 5,7 a 13,7) em uma população com CDI implantado exclusivamente para prevenção secundária.^4^ A segunda meta-análise, avaliando uma população mista de prevenção primária e secundária, revelou uma taxa anual agrupada de mortalidade por todas as causas de 9,0%. Esses achados destacam o risco significativo de mortalidade em pacientes com CC que possuem CDI, enfatizando a importância do monitoramento e manejo cuidadosos nessa população.

Além disso, a CC é reconhecida há muito tempo como uma cardiomiopatia altamente arritmogênica, com alta incidência de terapias apropriadas com CDI, com média de 24,8% ao ano,^5^ e uma taxa notável de tempestades elétricas de 9,1% ao ano, sublinhando a impressionante natureza arritmogénica da doença. Esta questão, no entanto, deve ser contextualizada devido à alta variabilidade na programação do CDI e nas estratégias implementadas para abordar a recorrência da taquicardia ventricular (TV), como medicamentos antiarrítmicos e ablação de TV. Existem evidências substanciais que associam terapias apropriadas, principalmente choques, ao aumento da mortalidade.

Nesta edição da ABC Cardiol, Pereira et al.,^5^ descrevem um estudo que avaliou pacientes com CC com CDI e os preditores de morte e terapias adequadas.^5^ A população do estudo foi predominantemente masculina (74%) com insuficiência cardíaca grave e 74% tiveram CDI implantado para prevenção secundária de morte súbita. A taxa de mortalidade global anualizada foi de 7,7%, semelhante às taxas de mortalidade para outras cardiomiopatias. Fração de ejeção do ventrículo esquerdo <30%, classe funcional IV e idade >75 anos foram associadas ao aumento da mortalidade após análise multivariada.

A taxa anual de terapia apropriada foi de 10% e 4,4% para tempestades elétricas, numericamente inferior aos dados agrupados apresentados na meta-análise anterior conduzida por Rassi et al.^6^ Este efeito pode ser parcialmente atribuído à tendência geral decrescente na mortalidade cardiovascular no Brasil, incluindo melhorias na programação do CDI, introdução de novos medicamentos para o tratamento da insuficiência cardíaca e acesso mais amplo à ablação de TV.

Curiosamente, nosso grupo publicou recentemente uma avaliação temporal de pacientes submetidos a implante de CDI antes e após a estruturação de Serviços de Arritmia e Eletrofisiologia em um sistema público terciário de saúde no Brasil.^7^ Além do principal achado de menor mortalidade para toda a população após o estabelecimento do atendimento especializado, mostramos um declínio progressivo nas taxas de recorrência de TV para pacientes com CC. Como resultado, no último período do nosso estudo, a disparidade nas taxas de TV/fibrilação ventricular (FV) entre pacientes chagásicos e não chagásicos não existia mais.

A estruturação do Serviço de Arritmia/Eletrofisiologia incluiu o acompanhamento rotineiro dos pacientes com CDI em clínicas especializadas, onde a programação dos dispositivos eletrônicos cardíacos era inteiramente realizada por equipe especializada, adotando práticas que podem reduzir a mortalidade. Este período também viu a introdução da ablação por cateter de TV, com um alto volume anual de ablações de TV chagásica. Foram introduzidas tecnologias inovadoras para o tratamento da CC, como o acesso epicárdico guiado por laparoscopia para prevenir lesões intra-abdominais de órgãos.^8^

Quanto ao benefício do implante de CDI em pacientes com CC, este permanece sendo um tema controverso. Embora dados recentes, como o manuscrito publicado nesta edição do ABC Cardiol por Pereira et al.,^5^ sugerem taxas de mortalidade semelhantes em comparação com outras cardiomiopatias, ainda faltam evidências diretas do benefício dos CDI em comparação com o tratamento médico ideal. Até que tenhamos evidências diretas derivadas de ensaios randomizados, esta questão permanecerá discutível.

Em resumo, a gestão da CC continua sendo um desafio complexo e em evolução, particularmente no que diz respeito ao uso de CDI. Embora estudos e meta-análises recentes indiquem altas taxas de eventos arritmogênicos e riscos significativos de mortalidade em pacientes com CC com CDI, o benefício direto dos CDI em comparação com o tratamento médico ideal ainda está em debate. A melhoria do atendimento especializado em arritmia tem mostrado resultados promissores, incluindo redução da mortalidade e declínio nas taxas de recorrência de TV, o que ressalta a importância do atendimento integral e especializado. No entanto, até que sejam obtidas evidências mais definitivas a partir de ensaios randomizados, o papel dos CDI na CC permanecerá sendo um tema controverso, necessitando de investigação contínua e avaliação clínica para otimizar estratégias de tratamento e melhorar os resultados dos pacientes.
