# Supplementary material for: Revisitando o Uso do CDI na Cardiomiopatia Chagásica: Evidências Atuais e Direções Futuras
Source: Arq Bras Cardiol. 2024 Aug 7;121(6):e20240423. [Article in Portuguese] doi: 10.36660/abc.20240423 (PMC11495634; doi:10.36660/abc.20240423)
Supplement: Translation

# Translation: Revisiting ICD Use in Chagas Cardiomyopathy: Current Evidence and Future Directions

**DOI:** 10.36660/abc.20240423i

**Authors:** André Carmo, Antônio Luiz Pinho Ribeiro

**Affiliations:** 1 Belo Horizonte MG Brazil Hospital das Clínicas and Faculdade de Medicina da Universidade Federal de Minas Gerais, Belo Horizonte, MG – Brazil

**Keywords:** Chagas Disease, Chagas Cardiomyopathy, Heart Failure, Stroke, Defibrillators, Implantable/trends

Chagas cardiomyopathy (ChC) is a chronic infectious heart disease that has recently been considered an infectious arrhythmogenic cardiomyopathy according to the HRS statement on this subject.^1^ After an acute phase, patients become chronically infected, displaying a wide range of presentations concerning heart disease, including heart failure, stroke, conduction abnormalities, atrial fibrillation, and ventricular arrhythmia.^2^ Sudden death is a mode of death of most patients with ChC, and implantable cardioverter-defibrillator (ICD) has been used as a preferred therapy, at least for secondary prevention, following recommendations similar to those of other cardiovascular diseases.^3^

All information on the use of ICD in ChC comes from observational studies and, regarding death rates, two meta-analyses have evaluated this topic in different populations. The first meta-analysis, conducted by our group, found an annual mortality rate of 9.7% (95% CI: 5.7 to 13.7) in a population with ICD implanted exclusively for secondary prevention.^4^ The second meta-analysis, evaluating a mixed population of primary and secondary prevention, revealed a pooled annual all-cause mortality rate of 9.0%. These findings highlight the significant mortality risk in patients with ChC who have ICDs, emphasizing the importance of careful monitoring and management in this population.

Additionally, ChC has long been recognized as a highly arrhythmogenic cardiomyopathy, with a high incidence of appropriate ICD therapies, averaging 24.8% per year,^5^ and a notable rate of electrical storms at 9.1% per year, underscoring the striking arrhythmogenic nature of the disease. This issue, however, must be contextualized due to the high variability in ICD programming and the strategies implemented to address ventricular tachycardia (VT) recurrence, such as antiarrhythmic drugs and VT ablation. There is substantial evidence linking appropriate therapies, primarily shocks, to increased mortality.

In this issue of ABC Cardiol, Pereira et al.^5^ describe a study evaluating ChC patients with ICDs and the predictors of death and appropriate therapies.^5^ The study's population was predominantly male (74%) with severe heart failure, and 74% had ICDs implanted for secondary prevention of sudden death. The annualized overall mortality rate was 7.7%, similar to mortality rates for other cardiomyopathies. Left ventricular ejection fraction <30%, functional class IV, and age >75 years were associated with increased mortality after multivariate analysis.

The annual rate of appropriate therapy was 10% and 4.4% for electrical storms, numerically less than the pooled data presented in the previous meta-analysis conducted by Rassi et al.^6^ This effect can be partially attributed to the overall decreasing trend in cardiovascular mortality in Brazil, including improvements in ICD programming, the introduction of new drugs for the treatment of heart failure, and wider access to VT ablation.

Interestingly, our group recently published a temporal evaluation of patients undergoing ICD implantation before and after the structuring of Arrhythmia and Electrophysiology Services in a tertiary public health system in Brazil.^7^ In addition to the main finding of lower mortality for the whole population after establishing specialized care, we showed a progressive decline in the rates of VT recurrence for patients with ChC. As a result, in the last period of our study, the disparity in rates of VT/ventricular fibrillation (VF) between Chagas and non-Chagas patients no longer existed.

The structuring of the Arrhythmia/Electrophysiology Service included the routine follow-up of ICD patients in specialized clinics, where the programming of cardiac electronic devices was entirely undertaken by specialized staff, adopting practices that can reduce mortality. This period also saw the introduction of VT catheter ablation, with an annual high volume of Chagas VT ablations. Innovative technologies for ChC treatment were introduced, such as laparoscopic-guided epicardial access to prevent intra-abdominal organ injury.^8^

Regarding the benefit of ICD implantation in patients with ChC, it remains a controversial topic. Although recent data, like the manuscript published in this issue of ABC Cardiol by Pereira et al.,^5^ suggest similar rates of mortality compared to other cardiomyopathies, direct evidence of the benefit of ICDs in comparison to optimal medical treatment is still lacking. Until we have direct evidence derived from randomized trials, this issue will remain debatable.

In summary, the management of ChC remains a complex and evolving challenge, particularly regarding the use of ICDs. While recent studies and meta-analyses indicate high rates of arrhythmogenic events and significant mortality risks in ChC patients with ICDs, the direct benefit of ICDs compared to optimal medical treatment is still under debate. The improvement of arrhythmia specialized care has shown promising results, including reduced mortality and a decline in VT recurrence rates, which underscores the importance of comprehensive and specialized care. However, until more definitive evidence is obtained from randomized trials, the role of ICDs in ChC will remain a controversial topic, necessitating continued research and clinical evaluation to optimize treatment strategies and improve patient outcomes.
